# Risk factors for the frequent attendance of older patients at community health service centers in China: a cross-sectional study based on stratified sampling

**DOI:** 10.1186/s12875-021-01575-w

**Published:** 2021-11-12

**Authors:** Nana Li, Juan Shou

**Affiliations:** General Practice Department, Zhongshan Hospital, Fudan University, 180 Fenglin Road, Xuhui District, Shanghai, 200032 China

**Keywords:** Community health service center, Frequent attender, Frequent attendance, China, Older patient

## Abstract

**Background:**

This study aimed to investigate the characteristics of frequent attenders (FAs) among older patients in Shanghai, China, and explore the associated factors.

**Methods:**

This cross-sectional study was conducted in six community health service centers in Shanghai, China, from August to December 2018 based on stratified sampling. On the basis of our preliminary study, FAs were defined as those attending at least four consultations in a month. A self-administered questionnaire was used to collect the clinicodemographic data of the participants. Social support, pain severity, depression, and anxiety were evaluated using the Social Support Revalued Scale, six-point Behavioral Rating Scale, Patient Health Questionnaire–9, and Generalized Anxiety Disorder Scale, respectively.

**Results:**

This study included 619 patients aged > 60 years. Among these patients, 155 (25%) were FAs to a community health service center, 366 (59.1%) had 1 or 2 chronic diseases, 179 (28.9%) had ≥3 chronic diseases, 313 (50.4%) opted for a family doctor service, and 537 (86.8%) chose a community health service center for the first consultation. The following were identified as independent risk factors for frequent attendance: widowed status, unmarried status, the presence of > 3 chronic diseases, first consultation at a community health service center, high medical expenses, frequent attendance of the spouses, long-term medication, the use of both traditional Chinese and Western medicine services, and depression.

**Conclusions:**

This study summarizes the characteristics of older FAs to community health service centers in China and identifies 10 risk factors significantly associated with frequent attendance.

## Background

Frequent attendance at community health service centers has resulted in a huge burden on the healthcare system, thus attracting increasing scientific attention in recent years [1]. Approximately 10% of the attending population has been reported to account for 40% of the total number of visits to general practitioners [2]. The definition of frequent attenders (FAs) varies substantially across studies [3]. Generally, FAs refer to a group of patients who visit healthcare providers more frequently than the general population, thereby using more medical resources with limited therapeutic effects [4]. FAs account for a substantial portion of visits to healthcare clinics, consume a large share of physician time, and increase the healthcare workload, ultimately contributing to higher expenses for healthcare services [5–7]. FAs are prone to suffer from more chronic physical and psychological diseases as well as medically unexplained symptoms and have poorer quality of life [8, 9].

The aging population with chronic diseases has become a global concern that demands increased attention [10]. The situation highlights the need for a deep understanding of the FAs among older patients. A retrospective study reported that older FAs to the emergency department in Singapore are at higher risk of mortality compared to the overall emergency department population [11]. A systemic review of European studies revealed that severe diseases and the need for treatment primarily drive the frequent attendance of older patients at primary care centers [12]. Moreover, a population-based longitudinal study in Germany provides evidence that older age, unemployment status, decreasing physical functioning, worsening health, and increasing physical diseases contribute to the onset of frequent attendance in middle-aged or older patients [13].

In response to the aging population and the imbalance in the medical service system in the country, China significantly revised its medical and health policies in 2009, aiming to provide equal basic medical and health services to all citizens [14]. In 2011, the family doctor contract program was introduced nationwide to encourage residents to prioritize the services provided by community health service centers when visiting a doctor. In 2015, the Chinese government issued guidelines for the establishment of a hierarchical medical service system. Medical institutions at all levels (third level, second level, and primary) provide services in accordance with designated functions. In China, with the recent advancement of the hierarchical diagnosis and treatment system, primary health services have assumed increasing responsibilities in the medical system, including the diagnosis and referral for common diseases, chronic disease management, rehabilitation, nursing, and other appropriate community medical services. Shanghai was one of the earliest cities in China to develop community health services. By the end of 2018, the number of residents participating in the “1 + 1 + 1” signing plan (one community health service center + one regional secondary hospital + one tertiary hospital) reached 6.66 million people, with a signing rate of 30%. Among these individuals, the signing rate of the key population, including the aged people was 54%.

Community health service centers are currently playing an increasingly important role in China. The coronavirus disease 2019 (COVID-19) outbreak reinforced the important role of community health service centers in screening, monitoring, and preventing COVID-19 and in providing other health services. However, the quality and efficiency of service provided at these centers still require improvement [15]. To our knowledge, limited studies have explored the frequent attendance of Chinese older patients at community health centers.

Therefore, this cross-sectional study investigated the frequent attendance of older patients at community health service centers in Shanghai, China, and unraveled the associated factors.

## Methods

### Definition of frequent attendance

The cross-sectional study was approved by the ethics committee of Zhongshan Hospital Affiliated with Fudan University, Shanghai, China. This study was conducted in accordance with the Declaration of Helsinki. To maximize the response rate, outpatients were targeted as study subjects during the survey period and they were selected onsite based on isometric sampling. For the convenience of self-assessment by older patients, FAs were defined as those with at least four visits to a primary care clinic in a month for the following reasons: no consensus has been reached regarding the definition of FA. In accordance with the definition proposed by Smits et al. [16], frequent attendance refers to visiting time among the top 10% of attendances during the study period. Hauswaldt et al. [17] identified patients with an intercontact interval of < 7 days as FAs by reviewing the electronic records at 123 general practice centers in Germany in a 10-year period. This indicates that a frequency of ≥4 visits per month can be defined as frequent attendance. In our previous retrospective study based on the medical records of patients from October 2014 to October 2017, we found that the lowest number of visits in the top 10% of the population each year was 53, 52, and 44 in 2015, 2016, and 2017, respectively, with an average of 4.14 visits per month [18]. Therefore, in this study, we defined FAs those with at least four visits to a primary care clinic in a month.

### Sample size

We conducted a preliminary study on 50 patients aged > 60 years who were selected from two community health service centers located in urban and suburban areas of Shanghai. The proportion of FAs was 24 and 26%. Using the average proportion of FA, *d* = 0.15p and α = 0.05, we calculated the sample size using the following formula:

$$\mathrm n=\frac{Z_\alpha^2\ast pq}{d^2}$$

Assuming a 20% dropout rate, the cross-sectional study required at least 614 patients.

### Sampling

There are 247 community health service centers in Shanghai, of which 79 are located in an urban area and 168 in the suburban area. This study adopted a stratified random-sampling method using a 1:1 ratio of urban and suburban health service centers. As a result, three urban community health service centers and three suburban community health service centers were selected using a random number generated by a computer program. The three selected urban community health service centers were Weifang Community Health Service Center in Pudong New District, Changfeng Community Health Service Center in Putuo District, and Fenglin Community Health Service Center in Xuhui District. The three selected suburban community health service centers were Xidu Community Health Service Center in Fengxian District, Huangdu Community Health Center District in Jiading District, and Community Health Service Center of Songnan Town in Baoshan District. The six community health service centers are located in the southeast and northwest of Shanghai. In this cross-sectional survey, sampling was started from the first older patient who attended the room one general clinic of each community health service center, with every other patient being selected. Patients not meeting the study criteria were not selected, and the next patient was then selected until the required sample size was reached.

### Inclusion and exclusion criteria

This study was successively conducted at six community health service centers from August to December 2018 and included a total of 619 patients. The inclusion criteria were an age of ≥60 years, willingness to participate in the questionnaire survey, and the provision of signed informed consent. The exclusion criteria were inability to communicate because of severe hearing or comprehension impairment and relatives or family members rather than patients visiting to the community health service centers.

### Data collection

A self-administered general questionnaire was used to collect demographic and clinical characteristic data of the patients. Specifically, it included sociodemographic parameters (i.e., age, sex, body mass index, education level, occupation, living condition, marital status, and self-perceived life satisfaction score), consultation-related parameters (i.e., first consultation choice, frequency of consultations at community health service centers and general hospitals, and the use of family doctors service), and health- and medication-related parameters (i.e., self-perceived health status score, pain score, hospitalization in the past 3 years, medical expenses, number of chronic diseases, and long-term medication), and psychological parameters (depression and anxiety).

Social support was evaluated using the Social Support Revalued Scale [19], which had 10 items and 3 dimensions (i.e., subjective support, objective support, and support utilization). A social support score of < 20 suggests weak social support; 20–30, modest social support; and > 30, satisfying social support. The severity of pain was assessed using a six-point Behavioral Rating Scale [20]. The score on this scale ranges from 1 to 6, with a higher score indicating more severe pain. The Patient Health Questionnaire–9 is a widely used tool for depressive disorder screening [21]. This scale includes nine items, with the score of each item ranging from 0 (*not at all*) to 3 (*almost every day*). The summed score ranges from 0 to 27 and is defined as follows: 0–4, no depression; 5–9, mild depression; 10–14, moderate depression; 15–19, moderately severe depression; 20–27, severe depression. The seven-item self-administered Generalized Anxiety Disorder Scale was used to detect generalized anxiety disorder [22]. The score of each item was 0 (*not at all*), 1 (*several days*), 2 (*> 50% of all days*), and 3 (*nearly every day*). The scores on this scale are divided into four categories, 0–4, 5–9, 10–14, and 15–21, that indicate *no*, *mild*, *moderate*, and *severe anxiety*, respectively.

After obtaining written informed consent from each participant, uniformly trained investigators conducted the face-to-face questionnaire survey. Each participant completed the questionnaire in 20 min under the guidance of one investigator. Regarding doubtful questions and options, the investigators provided adequate explanation to the participants. The questionnaire was collected on the spot and was numbered uniformly.

### Statistical analysis

All statistical analyses were performed using the SPSS software (version 18.0, SPSS Inc., Chicago, IL, USA). Quantitative data were expressed as means ± standard deviations, whereas categorical data were expressed as percentages or rates. A chi-square test together with stepwise logistic regression analysis was used to identify significant risk factors for frequent attendance of older patients. The significance was set at *p* <  0.05.

## Results

The study population was comprised 619 older patients who met the inclusion and exclusion criteria (mean age: 73.02 ± 8.05 years; men: 285 and women: 334). Of all enrolled patients, 59.1% (366/619) had one to two chronic diseases, 28.9% (179/619) had ≥3 chronic diseases, and 12.0% (74/619) had no chronic diseases. A total of 518 patients (83.7%) were on long-term medication (> 6 months). Table 1 shows other clinicodemographic characteristics of these patients.

**Table 1:** Demographic and clinical characteristics of all 619 patients

Variable	Group	Total number (N)	FAs (n(%))	*X*^2^	*P*-value
Gender	Male	285	61(0.21)	3.722	0.054
	Female	334	94(0.28)		
Age (years)	60~	228	46(0.20)	5.563	0.135
	70~	241	63(0.26)		
	80~	140	43(0.31)		
	90~	10	3(0.30)		
Education level	Elementary school and below	154	48(0.31)	4.425	0.219
	Middle high school	191	42(0.22)		
	High school or technical secondary school	170	39(0.23)		
	College and above	103	26(0.25)		
Marital status	Married	505	111(0.22)	15.249	0.002
	Widowed	108	42(0.39)		
	Divorced	3	1(0.33)		
	Unmarried	4	2(0.50)		
Living arrangement	Living alone	80	32(0.40)	10.954	< 0.01
	Not living alone	539	123(0.23)		
Medical insurance	No	14	1(0.071)	2.444	0.118
	Yes	605	154(0.25)		
Working status	Working	20	4(0.20)	0.28	0.597
	Retired	599	151(0.25)		
Monthly income (yuan)	< 3000	181	45(0.25)	6.232	0.044
	3000–5000	292	84(0.29)		
	> 5000	146	26(0.18)		
Self-evaluation of family relationship score	< 60	9	5(0.55)	20.457	< 0.01
	60–69	22	12(0.55)		
	70–79	56	20(0.36)		
	80–89	224	51(0.23)		
	≥90	308	67(0.22)		
Life satisfaction score	< 60	11	6(0.55)	15.635	< 0.01
	60–69	18	9(0.5)		
	70–79	65	22(0.34)		
	80–89	197	46(0.23)		
	≥90	327	72(0.22)		
Social support	Weak	35	16(0.46)	15.781	< 0.01
	Modest	256	75(0.29)		
	Satisfying	328	64(0.19)		
Self-perceived health score	< 60	57	30(0.52)	60.211	< 0.01
	60–70	111	44(0.39)		
	70–80	181	47(0.25)		
	80–90	158	25(0.15)		
	≥90	112	9(0.08)		
Number of chronic diseases	0	74	3(0.04)	77.405	< 0.01
	1–2	366	66(0.18)		< 0.01
	≥3	179	86(0.48)		
Monthly medical expenses (out-of-pocket cost, yuan)	< 100	243	19(0.08)	115.227	< 0.01
	100–199	109	15(0.14)		
	200–299	88	32(0.36)		
	300–399	54	33(0.61)		
	≥400	115	56(0.45)		
Long-term medication	No	101	5(0.05)	25.952	< 0.01
	Yes	518	150(0.29)		
Severity of pain	No	345	67(0.19)	35.448	< 0.01
	Light	121	28(0.23)		
	Modest	57	13(0.23)		
	Severe	96	47(0.49)		
Hospitalization in last 3 years	No	437	90(0.21)	15.648	< 0.01
	Yes	182	65(0.36)		
Depression	No	456	78(0.17)	58.091	< 0.01
	Yes	163	77(0.47)		
Anxiety	No	500	105(0.21)	25.581	< 0.01
	Yes	115	50(0.44)		

Of all 619 participants, 86.8% (537/619) attended a community health service center for the first consultation, whereas 13.2% (82/619) made their first visit to a general hospital or other medical institutions (Table 2). In total, 155 patients (25%) were FAs to a community health service center, whereas 289 (46.7%) seldom attended any general medical institution (Table 2). Of all patients, 50.4% (313/619) had signed the family doctor service, and 50.6% (313/619) attended fixed medical institutions (Table 2). Regarding the clinic type, 83.5% (517/619) patients consulted Western medicine clinics (Table 2), 10.5% (65/619) visited traditional Chinese medicine clinics, and 6% (37/619) visited both types of clinic (Table 2). Our preliminary study has reported that the frequency of consultation of the spouses might have influenced the consultation frequency of the patients; thus, in this study, we also studied the frequency of attendance of the spouses. The spouses of 79 patients were FAs to community health service centers, of whom 48 were FAs to the same community health service center as that visited by their spouse (Table 2).

**Table 2:** Results of chi-square tests on consultation-related factors

Variable	Group	Total number	FAs (n(%))	*X*^*2*^	*P*-value
First consultation choice	Community health service center	537	145(0.27)	8.309	< 0.01
	Other medical institutions	82	10(0.12)		
Frequency of consultations to at general hospitals	Seldom	289	56(0.19)	9.263	< 0.01
	≥1 time a year	110	33(0.30)		
	≥1 time a month	220	66(0.30)		
Consultation frequency of spouses (times/month)	< 4	540	107(0.20)	61.554	< 0.01
	≥4	79	48(0.61)		
Clinic type	Western medicine	517	117(0.23)	13.723	< 0.01
	Traditional Chinese medicine	65	20(0.31)		
	Both	37	18(0.49)		
Consultation to at fixed clinics	seldom	306	53(0.17)	20.632	< 0.01
	Often	149	44(0.29)		
	Always	164	58(0.35)		
Signed family doctor service	No	307	65(0.21)	4.854	0.028
	Yes	312	90(0.29)		

We used chi-square test to identify the clinicodemographic characteristics mentioned in the questionnaire that were significantly associated with frequent attendance among older patients. The results show that a total of 20 parameters were significantly associated with frequent attendance: marital status, living arrangement, monthly income, self-evaluation of family relationship score, life satisfaction score, social support, self-perceived health score, the number of chronic diseases, monthly medical expenses, long-term medication, pain severity, hospitalization in the past 3 years, anxiety, depression, first consultation choice, the frequency of consultations at general hospitals, frequent attendance of the spouses, clinic type, consultation at fixed clinics, and signed family doctor service (Tables 1 and 2).

We subsequently used 20 significant parameters in a stepwise multiple logistic regression analysis. We identified 10 independent risk factors for the frequent attendance of older patients (*p* <  0.05): widowed status (odds ratio [OR] = 3.308, 95% confidence interval [CI]: 1.886–5.802), unmarried status (OR = 13.004, 95% CI: 1.073–157.578), the presence of > 3 chronic diseases (OR = 5.420, 95% CI: 1.157–25.385), first consultation at a community health service center (OR = 2.593, 95% CI: 1.140–5.897), high monthly medical expenses (out-of-pocket cost; 200–299 yuan: OR = 3.028, 95% CI: 1.451–6.317; 300–399 yuan: OR = 7.323, 95% CI: 3.160–16.972; and ≥ 400 yuan: OR = 3.722, 95% CI: 1.839–7.533), frequent attendance of the spouses (OR = 8.861, 95% CI: 4.559–17.226), long-term medication use (OR = 3.988, 95% CI: 1.247–12.753), the use of both traditional Chinese and Western medicine services (OR = 2.679, 95% CI: 1.135–6.320), and depression (OR = 2.279, 95% CI: 1.387–3.746; Table 3).

**Table 3:** Identification of independent risk factors by a stepwise logistic regression analysis

Variable	Beta	SE	Wales	P	Exp (B)	95%CI	
						Upper	Lower
Marital status (vs. married status)			20.38	< 0.001			
Widowed	1.196	0.287	17.42	< 0.001	3.308	1.886	5.802
Divorced	−0.133	1.292	0.011	0.918	0.876	0.07	11.015
Unmarried	2.565	1.273	4.062	0.044	13.004	1.073	157.578
Monthly medical expenses (out-of-pocket cost, yuan, vs. < 100)			32.639	< 0.001			
100–199	−0.007	0.41	0	0.987	0.993	0.445	2.216
200–299	1.108	0.375	8.717	0.003	3.028	1.451	6.317
300–399	1.991	0.429	21.552	< 0.001	7.323	3.16	16.972
≥ 400	1.314	0.36	13.35	< 0.001	3.722	1.839	7.533
Number of chronic diseases (vs. 0)			17.645	< 0.001			
1–2	0.678	0.771	0.774	0.379	1.971	0.435	8.927
≥ 3	1.69	0.788	4.602	0.032	5.420	1.157	25.385
Consultation frequency of spouses (times/month, vs. < 4)	2.182	0.339	41.382	< 0.001	8.861	4.559	17.226
First consultation choice (vs. non-community health service center)	0.953	0.419	5.166	0.023	2.593	1.14	5.897
Long-term medication (vs. No)	1.383	0.593	5.441	0.02	3.988	1.247	12.753
Clinic type (vs. Western medicine)			7.564	0.023			
Traditional Chinese medicine	0.678	0.368	3.4	0.065	1.97	0.958	4.051
Both	0.985	0.438	5.06	0.024	2.679	1.135	6.32
Depression (vs. No)	0.824	0.254	10.558	0.001	2.279	1.387	3.746
Constant	−6.056	0.916	43.723	< 0.001	0.002		

## Discussion

Our study targeted patients aged > 60 years who attended community health service centers in Shanghai between August and December 2018. Our study enrolled 285 men and 334 men, with a mean age of 73.02 ± 8.05 years. Of these patients, 155 (25%) were FAs to a community health service center and 537 (86.8%) visited community health service center for the first consultation. We identified 10 independent risk factors of frequent attendance, including widowed status, unmarried status, the presence of > 3 chronic diseases, first consultation at a community health service center, high medical expenses, frequent attendance of the spouses, long-term medication use, the use of both traditional Chinese and Western medicine services, and depression.

In our study, FAs were defined as those with at least four visits to a primary care clinic in a month as we found the average monthly visits of patients aged > 60 years to a community health service center in Shanghai, China to be < 2. The lowest number of visits in the top 10% of the population per year was 53, 52, and 44 in 2015, 2016, and 2017, respectively, with an average of 4.14 visits per month [18]. In fact, there is currently no consensus on the definition of an FA [23]. Smits et al. [16] suggested that the proportion of FAs should be calculated based on different subgroups of at least three age categories per sex and proposed the definition of top 3% or 10% of enlisted patients in each 1-year age–sex group. A study conducted by Pasgaard et al. in a Danish population defined FAs as those with the upper quartile of the total number of measured consultations with a general practitioner over a period of 148 weeks [24]. However, these definitions might be more suitable for a retrospective study. On the other hand, Hauswaldt et al. [17] reviewed the electronic records from 123 general practice centers in Germany in a 10-year period and defined the patients with an intercontact interval of < 7 days as FAs. Thus, patients with a frequency of ≥4 visits per month can be defined as FAs. This definition is more convenient for self-assessment by older patients while participating in cross-sectional surveys on medical consultation in China. In fact, a previous cross-sectional study by Buczak-Stec et al. quantified FAs as the number of self-reported visits to general practitioners in the preceding 3 months as they found that a number of visits equal to or greater than the 90th percentile of all consultations corresponded to individuals who had ≥6 general practitioner consultations in the past 3 months in Germany [25]. To our knowledge, few studies have been conducted on FAs in China. Our definition of an FA might be of practical value in clinical practice and provide a preliminary reference for relevant studies on the use of community outpatient services in the future.

By investigating 54,849 participants aged 50–65 years in a Danish adult population, Jørgensen et al. found that FAs consulted their general practitioners 12.0 times, on average, a year [2]. Buczak-Stec et al. reported that the number of visits greater than or equal the 90th percentile of all consultations corresponded to individuals who had ≥6 general practitioner consultations in the past 3 months in Germany [25], which averages to two visits per month. The definition of FA in our study (four visits per month) is much higher than that used in other countries. This difference might have resulted from the different healthcare contexts of different countries. China reviewed its medical and health policies in 2009 to provide equal basic medical and health services to all citizens [1]. In 2011, the family doctor contract program was introduced nationwide to encourage residents to prioritize the services provided by community health service centers when visiting a doctor. In 2015, the Chinese government issued guidelines for the establishment of a hierarchical medical service system [2]. Medical institutions at all levels (third level, second level, and primary) provide services in accordance with designated functions. With the advancement of the hierarchical diagnosis and treatment system, the majority of the older patients intend to choose community health service centers for the first visit and 80–90% of health problems will be solved in primary healthcare institutions. In addition, China is committed to establishing primary healthcare services located within a 20-min walking distance in each neighborhood. Its advantages such as convenient consultation services, user-friendly family doctor contracting services, and large medical reimbursement rates have led to an increase in the frequency of patient consultations. In contrast to other countries, China has a huge population base, and its current health reform is still in its preliminary stage. The service scope of community health service centers includes the diagnosis and treatment of common diseases, dispensing, referral, chronic disease management, rehabilitation, nursing, health education, physical examination, and other required community medical services. The number of visits reported in this study also includes visits for the abovementioned services. Therefore, the patterns of attendance at primary healthcare centers in China are much higher than those in other countries.

Growing evidence has demonstrated that need factors are the predominant determinants of frequent attendance [26, 27]. To explore the factors associated with frequent attendance in more detail, we allowed the patients to freely express themselves and provide some feedback at a suitable time during the interview. We classified and summarized the factors that influenced frequent attendance into the following aspects: poor control of chronic diseases, the convenience of community hospitals, good doctor–patient relationship, more attention to their own health, self-perceived poor health, use of the family doctor signing policy, low social support, the influence of other people’s medical behaviors, the onset of acute illness, difficulty in moving, and economic reasons. The results of the stepwise multiple logistic regression analysis performed in our study revealed that an increasing number of chronic diseases, long-term medication use, the use of both traditional Chinese and Western medicine services, and high medical expenses were significantly associated with frequent attendance of older patients aged > 60 years, which is partly consistent with the results of European studies [12, 13, 25]. An Australian study also reported that chronic health condition and medication use were associated with the FA status [28]. It is possible that more chronic diseases and an increasing need for medications drive patients to visit community health service centers more frequently in order to promote health. This indicates that people are prone to use health services more frequently when suggested by their health care providers.

In contrast to a previous study, we identified a new factor that was significantly associated with FAs. We found that patients who use both Chinese and Western medicine services are more inclined to be an FA. Traditional Chinese treatment such as acupuncture and massage can be used in conjunction with or as an alternative to Western medicine. These treatment strategies usually require professional skill and are beyond the ability of patients or their relatives. Therefore, patients who used both Chinese and Western medicine services showed a tendency to require frequent medical treatments. In addition, because FAs reportedly experience greater health anxiety than non-FAs [8], the use of both traditional Chinese and Western medicine services results from the greater health anxiety in FAs in addition to multimorbidity. Therefore, general practitioners in community health service centers should master the skills associated with traditional Chinese medicine to meet the therapeutic demands of older patients.

Frequent visits to clinics inevitably generate sizable healthcare expenses [29]. Smits et al. concluded that FAs cause exceedingly high costs in not only primary care but also specialist care [5]. Furthermore, a recent study using data from remote communities in Taiwan indicated that FAs are characterized by higher costs related to prescriptions, therapeutics, and first visit than non-FAs [30].

In addition to physical diseases, FAs are more likely to have psychiatric illnesses than the general population [31]. Depression is one of the most common mental disorders in patients who are frequent users of healthcare services [32]. There is evidence that depressive episodes are associated with frequent attendance at occupational health primary care [6]. Moreover, depression may be an important determinant of frequent attendance of older patients at healthcare centers [33]. Consistently, we found that in older patients in China, depression was an independent risk factor for frequent attendance at community health service centers. Furthermore, more depressive disorders, including dysthymia, anxiety, and somatoform disorders, have been reported in FAs than in non-FAs [8]. This suggests that general practitioners should pay more attention to older patients with concomitant depressive syndromes and provide timely and effective interventions. Cognitive behavioral therapy is able to relieve depression in FAs in primary care [34]. Further efforts are needed to develop more tailored and effective therapies against depression in FAs.

A previous study found that divorce is significantly associated with frequent attendance of FAs [23]. Conversely, another study revealed that the civil status was not associated with frequent attendance [33]. The present study identified three factors associated with the spouses of the FAs that influenced the frequent attendance of the older patients: widowed status, unmarried status, and frequent attendance of the spouses. We speculated that the high frequency of the attendance of widowed and unmarried patients might be associated with the mental and psychological state of living alone. The influence of spouses on FAs suggests that the FAs should be managed as a family unit for precise diagnosis and adequate treatment of these patients. These paradoxical results may be attributed to different definitions of FAs, limited sample size, and various analytical tools used in these studies. Further studies and more in-depth analyses are warranted to further decipher the relationships between the civil status and frequent attendance of older patients.

Our study has some limitations. This cross-sectional study explored the factors associated with frequent attendance but could not determine the presence of any causal relationship. Moreover, the sample size was small. Further thorough investigations on the FAs among older patients should be performed using a larger sample size in the future. In addition, general practitioners in community health service centers should pay close attention to the social, physical, and psychological needs of the FAs among older patients, thus developing more cost-effective health interventions for these patients.

## Conclusions

This study unravels the characteristics of frequent attendance of older patients at community health service centers in China and identifies 10 independent risk factors of frequent attendance (widowed status, unmarried status, the presence of > 3 chronic diseases, first consultation at a community health service center, high medical expenses, frequent attendance of the spouses, long-term medication use, the use of both traditional Chinese and Western medicine services, and depression). Our findings expand the understanding of frequent attendance of older patients and will help guide community health service centers in improving the quality of healthcare in China.

## Data Availability

All data generated or analyzed during this study are included in this manuscript.

## Abbreviation

FA: Frequent attender
